# Work-Family Organizational Support as a Predictor of Work-Family Conflict, Enrichment, and Balance: Crossover and Spillover Effects in Dual-Income Couples

**DOI:** 10.5964/ejop.v16i1.1931

**Published:** 2020-03-03

**Authors:** Alessandro Lo Presti, Monica Molino, Federica Emanuel, Alfonso Landolfi, Chiara Ghislieri

**Affiliations:** a Dipartimento di Psicologia, Università degli Studi della Campania “Luigi Vanvitelli”, Caserta, Italy; b Dipartimento di Psicologia, Università degli Studi di Torino, Turin, Italy; c Dipartimento di Filosofia e Scienze dell’Educazione, Università degli Studi di Torino, Turin, Italy; Department of Psychology and Counselling, Webster University Geneva, Geneva, Switzerland

**Keywords:** dual-income couple, work-family balance, family-life satisfaction, work-family organizational support, crossover effect, spillover effect

## Abstract

Dual-income families are challenged by several issues in terms of conciliation between the working environment, the family context and the management of children. This paper, consistently with spillover and crossover hypotheses, aimed at examining the intermediate role of work-family balance, linking on work-family organizational support, work-to-family enrichment and conflict as predictors, and on family-life satisfaction of dual-income families’ both partners as final outcomes. It was expected that work-family organizational support would be related to lower work-to-family conflict and higher enrichment and, through them, with higher work-family balance; moreover, a positive association between work-family balance and family-life satisfaction of both partners was assumed. 390 double-income heterosexual couples participated in our study; 76.2% of the couples were parents. Structural equation modelling results showed that work-family balance was negatively predicted by work-to-family conflict and positively predicted by work-to-family enrichment. Furthermore, work-family organizational support positively predicted work-to-family enrichment, which also mediated its effect on work-family balance. Crossover and spillover effects were also confirmed, given that positive associations between work-family balance and family-life satisfaction of both partners were found. Implications for future research and organizational interventions aimed at both improving work-family balance and promoting greater satisfaction in family life are discussed.

Despite the persistence of the economic crisis that has severely affected many countries and caused widespread unemployment (Giunchi, Emanuel, Chambel, & Ghislieri, 2016), dual-income families are still common in contemporary Europe (Fahlén, 2015). This is perhaps due also to the increased participation of women in the labour market. In Italy, dual income couples represent 32% of all families (Instituto Nazionale die Statistica [ISTAT], 2017); a rate that is growing, especially in northern and central regions. Similar trends and statistics can be observed in several Western countries, demonstrating how dual-income are becoming the dominant breadwinner kind of families, with predictable implications in terms of social welfare, the organization of work, social life and so on. According to the US Bureau of Labour Statistics (2018), for example, from every 100 American families, 80.5% have at least one family member who is in employment and 48.3% of them are dual income households, where both partners work.

With this backdrop, employees and employers are interested in measures that may promote work-family balance: employees look for organizations that can facilitate their being a member of a family where both partners work and thus have a complex series of work-family needs. Employers are also aware of this social shift and the consequent needs, and respond by trying to offer better working conditions in terms of flexibility, parental and family leave, etc.

Typing “work-family best place to work” on the Google search engine returns 128 million results, with a series of rankings within the top results: Business Insider, Great Place to Work, Money – Time Magazine, Indeed.com, Forbes and so on. It can be argued that organizations are nowadays increasingly conscious that fostering employees’ work-family balance may provide benefits. As a result, they publicize their change of attitude and practice, applying for rankings and legitimating themselves as work-family friendly organizations to employees, customers and suppliers. This involves offering formal work-family support and also promoting a shift in organizational culture and climate, which results also in informal work-family support.

We may usually consider a wide array of options and benefits designed to alleviate the burden of managing work and family (e.g., supportive leave policies, company kindergartens, flexitime, etc.) as sitting within the label of formal work-family support. It is well known, however, that a series of “organizational phenomena that are not mandated or proscribed by formal organizational policies or programs” (Behson, 2005, p. 488) can also have a fundamental role in justifying and supporting the implementation and legitimization of these benefits, namely informal work-family support. In fact, it can often be the case that official policies do not have the intended effect as they do not bring with them an equal change in organizational culture and climate (Allen, 2001). To explain, employees may fear the negative repercussions associated with benefiting from these allowances (e.g., being highlighted as workers with low commitment or a lower desire for upward mobility) and, consequently, they decree their failure.

One of the most popular concepts in this area is work-family culture, defined as “the shared assumptions, beliefs, and values regarding the extent to which an organization supports and values the integration of employee’s work and family lives” (Thompson, Beauvais, & Lyness, 1999, p. 394). In this analysis, Thompson et al. (1999) outlined three main factors of work-family culture: work-family organizational support (henceforth WFOS), career consequences related to the use of work-family programs and working time expectations hindering the fulfilment of the family role. Among them, the most prominent is WFOS, which is the extent to which employees perceive their managers and the organization as being supportive in helping them find a balance between work and family domains. WFOS has been extensively linked with reducing work-family conflict (Mauno, 2010; Mauno, Kinnunen, & Pyykkö, 2005) and increasing work-family enrichment (Lo Presti & Mauno, 2016; Peeters, Wattez, Demerouti, & de Regt, 2009), while its association with work-family balance has received less empirical attention (Carlson, Grzywacz, & Zivnuska, 2009). Work-family balance can be considered a novel variable in the work-family research field that is clearly distinct from conflict and enrichment. It is increasingly used in research on work-family interface and requires further attention and empirical investigation (Grzywacz & Carlson, 2007; Orkibi & Brandt, 2015).

Starting from these considerations and building on the Spillover-Crossover Model (SCM; Bakker & Demerouti, 2013), the present paper aims at examining the intermediate role of work-family balance. It links WFOS, work-to-family enrichment and conflict as predictors on one side, and the family-life satisfaction of dual-income families’ partners as final outcomes on the other side. It is predicted that WFOS will be related to lower work-to-family conflict and higher enrichment and, through them, to higher work-family balance; this latter will finally lead to higher family-life satisfaction for both partners. Thus, this paper addresses four main shortcomings in current literature: a) it examines the role of WFOS as a job resource that can impact on both work-family conflict and enrichment, b) it disentangles the differential effects of conflict, enrichment, and balance on family-life satisfaction, c) it examines the relationship between work-family balance and family-life satisfaction of both partners of dual-income families and d) it tests the SCM in the Italian context, also integrating the concept of work-family balance. From a practical standpoint, organizational interventions aimed at fostering WFOS (e.g., training supervisors to be sensitive to their subordinates’ needs) and developing work-family balance (e.g., training employees to improve their ability to balance work and familial demands and responsibilities) could also improve satisfaction with family life.

## Theoretical Foundation

According to Grzywacz and Carlson’s (2007) definition, work-family balance is the “accomplishment of role-related expectations that are negotiated and shared between an individual and his/her role-related partners in the work and family domains” (p. 458). Later, several scholars called for a differentiation between this construct and both work-family enrichment and conflict; Grawitch, Maloney, Barber, and Mooshegian (2013) stated that work-family balance is “distinct from conflict and facilitation [i.e., enrichment], as it is concerned with global perceptions of how one is allocating resources among domains rather than how domains are conflicting with or facilitating each other” (p. 277). Additionally, Casper, Vaziri, Wayne, DeHauw, and Greenhaus (2018) argued that “our findings and other recent evidence […] suggest that balance is distinct from conflict and enrichment, but how these variables relate to each other is not well-understood” (p. 201). They also called for further research in order to differentiate these constructs.

Work-family conflict is considered “a form of inter-role conflict in which the demands of work and family roles are incompatible in some respect so that participation in either the work or family role is more difficult because of participation in the other role” (Greenhaus & Beutell, 1985, p. 77), while work-family enrichment is considered “the extent to which experience in one role improves the quality of the life in the other role” (Greenhaus & Powell, 2006, p. 73).

The present study builds on the SCM of Bakker and Demerouti (2013) to explain the complex interplay between WFOS (i.e., a job resource) and work-family conflict and enrichment in terms of the family-life satisfaction of both the focal person (i.e., the spillover effect) and his/her partner (i.e., the crossover effect). This is done through the mediation of work-family balance.

The spillover theory can be traced back to about 40 years ago, when Kanter (1977) argued that “occupations contain an emotional climate as well that can be transferred to family life. A person’s work and relative placement in an organization can arouse a set of feelings that are brought home and affect the tenor and dynamics of family life” (p. 47). As for crossover, Westman (2001) defined it as “the reaction of individuals to the job stress experienced by those with whom they interact regularly” (p. 717). It has been mainly studied with reference to work-family interference to understand how experiences lived in the work and family domains “cross over” from a person to his/her partner (Parasuraman & Greenhaus, 2002).

More recently, the SCM (Bakker & Demerouti, 2013) combined both the spillover and crossover theories, proposing that work-related experiences first spill over to the home domain, and then cross over to the partner through social interaction. The SCM is based upon the Job Demands-Resources model (Bakker & Demerouti, 2008) which proposed that every job may be described in terms of working conditions, namely job demands and job resources. According to the SCM’s spillover mechanisms, while job demands are hypothesized to spill over into the home domain resulting in higher work-family conflict, job resources should enable higher work-family enrichment. In terms of SCM’s crossover mechanisms, it is expected that an individual’s positive or negative experience will cross over to her/his spouse both directly (i.e., emotional contagion) and indirectly (i.e., by interpersonal exchange). We propose that work-family balance, as defined by Grzywacz and Carlson (2007), can be considered to be a proxy of interpersonal exchange. This was also theorized by Bakker and Demerouti (2013), as it emphasizes the accomplishment of role-related expectations between the couple’s partners. This is consistent with authors (Casper et al., 2018) who stressed the relational component of Grzywacz and Carlson’s (2007) work-family balance definition, in opposition to others (Valcour, 2007).

## Study Hypotheses

As for the interplay between work-family enrichment, conflict and balance, Carlson et al. (2009) believed this latter variable to be more global in perspective than conflict and enrichment. Greenhaus and Allen (2011) suggested that while work-family conflict and enrichment are linking mechanisms that specify how one role affects another, work-family balance reflects an overall, holistic appraisal. Later, Casper et al. (2018) argued that work-family “balance measures are empirically distinct from and predict job and family variables above conflict and enrichment” (p. 197), thus suggesting that differentiating balance from conflict and enrichment is worthwhile.

As for empirical evidence, Carlson et al. (2009) research on work-family balance showed a positive association with work-family enrichment and a negative one (albeit weaker) with work-family conflict. Greenhaus, Ziegert, and Allen (2012) observed that work-family balance was negatively associated with work-family interference and later, Grawitch et al. (2013) found that work-family balance was predicted by both work-life conflict and enrichment. It appears that facing difficulty in meeting the demands of one role (e.g., family) because of the conflict (i.e., work-family conflict) deriving from the demands of the other role (e.g., work) could impair the ability to accomplish role-related expectations and thus work-family balance; from a complementary point of view, the improvement (i.e., work-family enrichment) of the quality of life in one role (e.g., family) in consequence of experiences in the other role (e.g., work) might have a beneficial influence on work-family balance. Based on the above-mentioned considerations and evidence, it is expected that:

H1a: Work-to-family conflict will be negatively associated with work-family balance.

H1b: Work-to-family enrichment will be positively associated with work-family balance.

As anticipated earlier, it is possible that other factors apart from work-to-family conflict and enrichment may affect work-family balance (Carlson et al., 2009). Among job resources, a preeminent role can be attributed to WFOS. Consistently with hypotheses 1 and 2, we suppose that WFOS will have a positive relationship with work-family balance, mediated by work-to-family enrichment and conflict. In detail, in previous studies (e.g., Carlson & Perrewé, 1999; Cohen & Wills, 1985; Seiger & Wiese, 2009), social support from different sources, including from organizations and supervisors, showed a positive association with work-family conflict. However, a meta-analytic review (Michel, Kortba, Mitchelson, Clark, & Baltes, 2011) demonstrated that social support received in both work and family domains had a negative association with work-to-family conflict. Other researchers (Frone, Yardley, & Markel, 1997; Glass & Estes, 1997; Greenhaus & Beutell, 1985; Greenhaus & Parasuraman, 1999) examined the association between social support and work-family conflict, considering it to be an important antecedent of both family-to-work and work-to-family conflict. It was also found that support from supervisors and co-workers could decrease work-family conflict (Bellavia & Frone, 2005). In recent years, Kossek, Pichler, Bodner, and Hammer (2011) and Selvarajan, Cloninger, and Singh (2013) found that workplace social support was significantly negatively associated with work-to-family conflict. Summing up, consistent with the SCM positive spillover hypothesis, it can be expected that WFOS will diminish work-family conflict. This will occur because the availability of informal work-family support from co-workers, supervisors and the organization could be helpful in either removing or coping with potential job aspects that are detrimental to the individual’s work-family interface. Accordingly, we expect the following:

H2a: WFOS will be negatively associated with work-to-family conflict.

Evidence about the association between WFOS and work-family enrichment is sparse compared to that pertaining to work-family conflict. Peeters et al. (2009) found a positive relationship between work-to-family enrichment and work-family culture and a mediation of work-to-family enrichment in the relationship between work engagement and burnout. Later, Lo Presti and Mauno (2016) found that WFOS positively predicted work-to-family enrichment, over and above work-family barriers (i.e., the negative side of work-family culture). From a theoretical point of view, WFOS can be considered as a specific form of organizational support (Rhoades & Eisenberger, 2002). It is well-known that when people feel supported by their organizations, they tend to maximize resources at work (Rhoades & Eisenberger, 2002) which, in turn, may have positive effects on their private and family lives. Put differently, people who perceive a higher WFOS will be more likely to bring positive resources obtained in the work domain to their family life. A higher WFOS allows individuals to feel that their socio-emotional needs are satisfied in their job role resulting in a higher performance and better mutual behaviour (i.e., expansion hypothesis; Marks, 1977). This positive psychological experience may then be applied to family life (i.e., work-to-family enrichment). Thus, consistent with the SCM positive spillover hypothesis, it is expected that the availability of family-friendly informal support within an organization could provide the individual with a greater number of resources that, in turn, can be transferred from the work to the family domain:

H2b: WFOS will be positively associated with work-to-family enrichment.

Ferguson, Carlson, Zivnuska, and Whitten (2012) argued that “very little is known about the role of social support in relation to work-family balance and the accomplishment of role-related expectations that it embodies” (p. 300). Later in their study, they observed a positive relationship between co-worker social support and work-family balance. Additionally, Greenhaus et al. (2012) hypothesized that work-family interference (i.e., a concept similar to work-family conflict) would mediate the relationship between family-supportive supervision and work-family balance, finding a total mediation. Later, Talukder, Vickers, and Khan (2018) found that among different types of support offered in a job, having empathic supervisors (i.e., accommodating, able to listen to employees and be sensitive to their personal and family needs) may play an important role in helping employees achieve a better work-life balance. Summing up, we can argue that work-family sensitive sources of social support are beneficial in integrating work and family roles. This is also consistent with the SCM which stated that the effects from job resources and demands on interpersonal exchange (i.e., work-family balance) would be mediated by work-family conflict and enrichment. Taking into consideration what was discussed in relation to hypotheses 1 and 2, we can also hypothesize that:

H3a: WFOS will be positively associated with work-family balance.

H3b: The association between WFOS and work-family balance will be mediated by work-to-family conflict.

H3c: The association between WFOS and work-family balance will be mediated by work-to-family enrichment.

As stated above, the experience of WFOS will result in increased work-family balance through the joint processes of increased work-to-family enrichment and reduced work-to-family conflict. Therefore, individuals will encounter more positive experiences and feel more stimulated and inspired at work. Probably, reflecting the SCM, they will also continue to enjoy the resources and benefits acquired after returning home (i.e., the spillover hypothesis), displaying greater happiness, enthusiasm and confidence in interacting with their partner and children. This could in turn increase family satisfaction and consequently (according to the crossover hypothesis) partner’s satisfaction.

Regarding available evidence about spillover effects, several scholars reported significant associations between work-family balance and improved marital and family satisfaction levels (Allen, Herst, Bruck, & Sutton, 2000). This in turn resulted in improved family performance (Frone et al., 1997). Milkie and Peltola (1999) found a positive association between work-family balance and conjugal happiness and even with a more balanced distribution of domestic work. Similar results have been found by Clarke, Koch, and Hill (2004) who reported that a greater balance between work and family resulted in improved marital satisfaction and time spent in domestic activities. Therefore, considering these results and this theory, we hypothesize that higher levels of balance between work and family domains could have an association with positive outcomes in family life. Therefore:

H4a: Work-family balance will be positively associated with the focal person’s family-life satisfaction.

H4b: The association between WFOS and the focal person’s family-life satisfaction will be mediated by work-family balance.

As for crossover effects, though previous cross-sectional studies examined the crossover of life satisfaction (Demerouti, Bakker, & Schaufeli, 2005), most of them have focused on negative experiences, neglecting the positive side of work-family balance crossover. Some scholars have found that positive experiences such as life satisfaction or happiness can be exchanged between partners (Demerouti et al., 2005; Rodríguez-Muñoz, Sanz-Vergel, Demerouti, & Bakker, 2014) and emotional contagion literature has been used to explain the crossover of positive feelings (Sanz-Vergel & Rodríguez-Muñoz, 2013). More recently, Ferguson et al. (2012) discovered a positive association between work-family balance and the partners’ family satisfaction levels. According to the SCM, the improvement of interpersonal exchange, proven by the ability to accomplish role-related expectations by the focal-person (i.e., work-family balance), could foster positive reactions and attitudes in his/her spouse, resulting in heightened family-life satisfaction. Based on extant literature, we hypothesize that:

H5a: Work-family balance will be positively associated with partner’s family-life satisfaction.

H5b: The association between WFOS and partner’s family-life satisfaction will be mediated by work-family balance.

**Figure 1:**
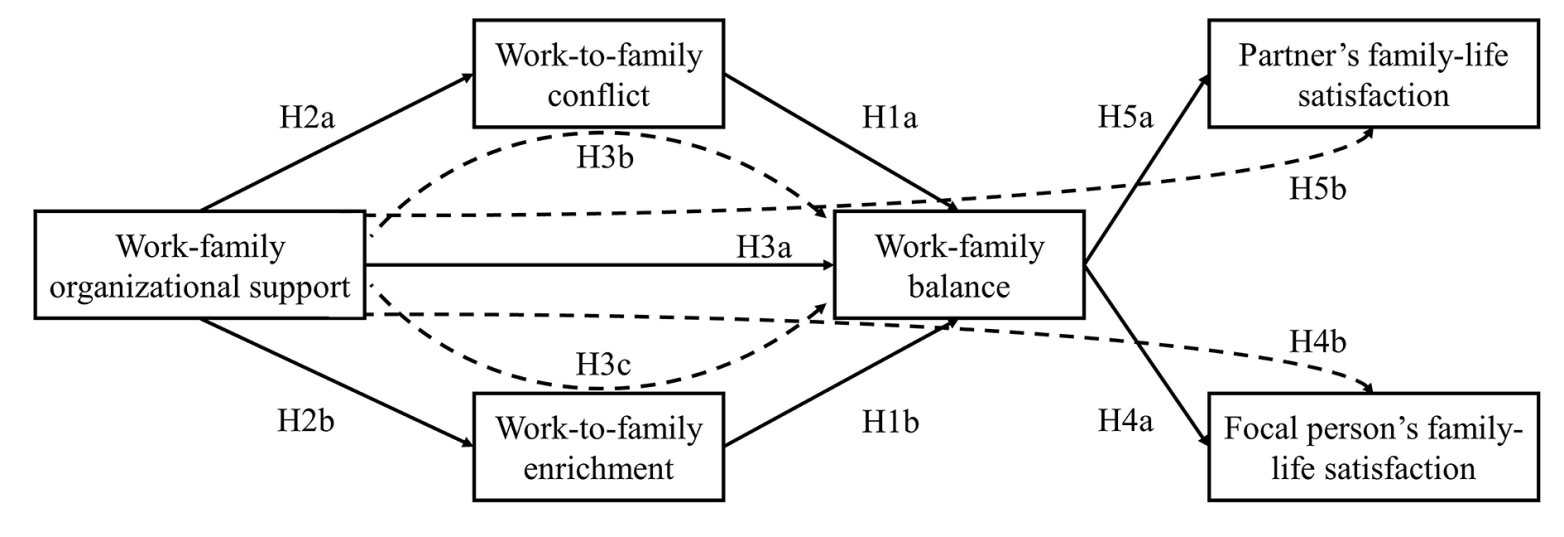
Research model (dotted lines = mediation).

Figure 1 depicts the theoretical model and study hypotheses.

## Method

### Participants

A total of 390 heterosexual dual-income couples participated in our study. The focal persons’ mean age was 43 years (*SD* = 9.39), with the range being between 25 and 67 years (partners’ ages were not considered). Among focal persons, 216 (55.4%) were female and 174 (44.6%) were male.

Regarding the educational levels of focal persons, 32 (8.2%) held an elementary/junior high school certificate, 169 (43.3%) a high school diploma and 189 (48.5%) a university degree or higher. As for their partners, 51 (13.1%) held an elementary/junior high school qualification, 207 (53.1%) a high school diploma and 132 (33.8%) a university degree or higher.

Regarding the employment statuses of focal persons, 337 (86.4%) held a permanent contract, 42 (10.8%) a temporary one, 10 (2.6%) held other statuses (e.g., freelance; 1 missing value equalling 0.3%). As for their partners, 242 (62.5%) held a permanent contract, 86 (22.1%) a temporary one, 50 (12.8%) had other statuses (e.g., self-employed; and 12 had a missing value equating to 2.6%).

As for the 390 couples, 297 of them (76.2%) were also parents. The average number of children was 1.91 (*SD* = 0.78) with number of children ranging from 0 to 5. A total of 157 couples (40.5%) had both members with an elementary/junior high school qualification or high school diploma, 87 couples (22.3%) had the focal person with a university degree or higher and his/her partner with a lower level of education, 43 couples (11.0%) had the focal person with an elementary/junior high school qualification or high school diploma and his/her partner with a university degree or higher, 86 couples (22.1%) had both members with a university degree or higher, 16 couples (4.1%) indicated other educational levels or missing data. In 218 couples (67.3%) both members had a permanent contract, in 73 couples (22.5%) the focal person had a permanent contract and his/her partner another contract, 21 couples (6.5%) had the focal person with a not-permanent contract and his/her partner with a permanent one, and in 12 couples (3.7%) both members had another type of contract.

### Procedure

This study used a self-report questionnaire to involve human beings in a research project conducted in line with the rules of the national law on data handling followed by the University of Campania and the University of Turin (Italy). Participants were all healthy adult subjects who participated anonymously and the procedure did not involve medical treatment nor cause any kind of discomfort; therefore, further ethical consent was not required, according to the Institutions. The Helsinki Declaration (World Medical Association, 2001) and the Italian laws of data protection (Legislative Decree No. 196/2003) were adhered to in this research. Involvement in the research was anonymous, voluntary and not rewarded. Information about the study aims, anonymity, voluntary participation and the treatment of data was provided along with instructions for filling out the questionnaire. When agreeing to fill out the questionnaire, all study participants provided their informed consent. Participants were contacted within participant organizations by trained researchers. Once availability to participate was obtained, participants received an envelope containing the questionnaire. It consisted of several sections where the final one was reserved for the focal person’s spouse. s A letter of presentation and instructions for both partners were also included; they were asked to fill in those sections dedicated to them separately, without making any interpersonal comparisons. Once the questionnaire was filled in, participants were asked to seal and return the envelope to the trained researchers either directly or indirectly through sealed urns left within organizations.

### Measures for Focal Persons

#### Work-Family Organizational Support 

Work-Family Organizational Support (WFOS; Thompson et al., 1999; Italian version by Lo Presti, Spagnoli, Ghislieri, & Pluviano, 2017) refers on the perceived easiness and supportiveness of balancing work and family within the organization, managerial empathy toward employees’ conciliation needs, etc. It included nine items (e.g., “Higher management in this organization encourages supervisors to be sensitive to employees’ family and personal concerns”). Participants used a 5-point Likert scale ranging from 1 = completely false to 5 = completely true. Cronbach alpha in this study was .86. Scores ranged between 1 and 5.

#### Work-to-Family Conflict

Work-to-family conflict (Netemeyer, Boles, & McMurrian, 1996; Italian version by Colombo & Ghislieri, 2008) measured how often the fulfillment of the work role interferes with the fulfillment of the family role using five items (e.g., “Due to work-related duties, I have to make changes to my plans for family activities”) with a frequency scale from 1 = never to 6 = always. Cronbach alpha was .90. Scores ranged between 1 and 6.

#### Work-to-Family Enrichment

Work-to-family enrichment (Carlson, Kacmar, Wayne, & Grzywacz, 2006; Italian short version by Ghislieri, Martini, Gatti, & Colombo, 2011) measured the improvement of family life quality because of experiences in the work life through three items (e.g., “At work I feel positive emotions and this helps me to be a better family member”) with a 5-point scale from 1 = completely disagree to 5 = completely agree. Cronbach alpha was .92. Scores ranged between 1 and 5.

#### Work-Family Balance

Work-family balance (Carlson et al., 2009) comprised six items (e.g., “I am able to accomplish the expectations that my supervisors and my family have for me”) with a 5-point Likert scale from 1 = completely disagree to 5 = completely agree in order to detect how much a person is able to meet role-related expectations in both the work and family spheres. Cronbach alpha was .94. Scores ranged between 1 and 5.

### Measures for Focal Persons and Their Partners

#### Family-Life Satisfaction

Family-life satisfaction (adapted from the satisfaction with life scale, Kobau, Sniezek, Zack, Lucas, & Burns, 2010) measured how much the respondent is satisfied with his/her own family life using five items (e.g., “In most ways my family life is close to my ideal”) with a 7-point Likert scale from 1 = completely disagree to 7 = completely agree. Cronbach alpha was .94 for focal persons and .93 for their partners. Scores ranged between 1 and 7.

### Control Variables

Focal person’s sex, number of children and employment contract for both the focal person and his/her partner were inserted as control variables.

### Data Analysis

Missing values (0.35%) were replaced from Expectation-Maximization technique. Descriptive statistics and correlations were calculated through IBM SPSS 24 in order to investigate associations between variables. Measurement and structural equation models were analyzed through Lisrel 9.3; the method of estimation was Maximum Likelihood.

We applied the item parceling technique to create parcels of items belonging to a same variable (Little, Cunningham, Shahar, & Widaman, 2002). Item parceling was applied to all constructs (statistics are available under request from the first author): work-family organizational support (3 parcels for 9 items), work-to-family conflict (3 parcels for 5 items), work-to-family enrichment (3 parcels for 3 items), work-family balance (3 parcels for 6 items), satisfaction with family life (3 parcels for 5 items), and partner’s satisfaction with family life (3 parcels for 5 items).

As for goodness-of-fit indices, we referred to chi square difference test to assess differences between nested models; moreover, according to literature (Cudeck & Browne, 1993; Hooper, Coughlan, & Mullen, 2008; Hoyle, 1995; Hu & Bentler, 1999) we considered the Comparative Fit Index (CFI), the Goodness of Fit Index (GFI), the Standardized Root Mean square Residual (SRMR), and the Root Mean Square Error of Approximation (RMSEA).

Since we used a self-report questionnaire, we addressed response and common method biases following suggestions by Podsakoff, MacKenzie, Lee, and Podsakoff (2003). Hence, into the questionnaire scales were graphically separated from each other and items were randomly inserted; furthermore, in order to reduce commonalities and anchoring effect biases, we measured predictor and criterion variables using different scale endpoints and formats.

## Results

Table 1 shows results of descriptive and correlation analyses.

**Table 1:** Descriptive Statistics and Zero-Order Correlations

Variable	*M*	*SD*	1	2	3	4	5	6	7	8	9	10
1. Sex^a^	-	-	-									
2. Number of children	1.46	1.06	-.10	-								
3. Employment contract^b^	-	-	-.15**	.24***	-							
4. Partner’s employment contract^b^	-	-	.17**	.03	-.08	-						
5. Work-family organizational support	2.94	0.75	.01	-.06	-.13*	.00	-					
6. Work-to-family conflict	2.87	1.18	-.02	-.02	.01	.00	-.08	-				
7. Work-to-family enrichment	3.27	1.15	.01	-.03	-.10	-.07	.51***	.02	-			
8. Work-family balance	3.77	0.94	.02	-.09	-.05	.01	.48***	-.11*	.54***	-		
9. Family-life satisfaction	4.93	1.53	.01	.05	.00	.03	.38***	-.21***	.36***	.60***	-	
10. Partner’s family-life satisfaction	5.10	1.43	-.00	.09	-.00	.05	.30***	-.13**	.28***	.50***	.73***	-

^a^1 = woman, 0 = man. ^b^1 = permanent, 0 = not permanent.

*p < .05. **p < .01. ***p < .001.

Work-family organizational support showed a positive correlation with work-to-family enrichment (*r* = .51, *p* < .001), work-family balance (*r* = .48, *p* < .001), family-life satisfaction (*r* = .38, *p* < .001), and partner’s family-life satisfaction (*r* = .30, *p* < .001). Work-to-family conflict showed a negative correlation with work-family balance (*r* = -.11, *p* < .05), family-life satisfaction (*r* = -.21, *p* < .001), and partner’s family-life satisfaction (*r* = -.13, *p* < .01). Work-to-family enrichment showed a positive correlation with work-family balance (*r* = .54, *p* < .001), family-life satisfaction (*r* = .36, *p* < .001), and partner’s family-life satisfaction (*r* = .28, *p* < .001). Work-family balance showed a positive correlation with family-life satisfaction (*r* = .60, *p* < .001), and partner’s family-life satisfaction (*r* = .50, *p* < .001). Finally, family-life satisfaction positively correlated with partner’s family-life satisfaction (*r* = .73, *p* < .001).

As for control variables, being a woman meant a lower likelihood of having a permanent employment contract (*r* = -.15, *p* < .01) and a higher likelihood of having a partner with a permanent contract (*r* = .17, *p* < .01). The number of children positively correlated with permanent employment contract (*r* = .24, *p* < .001), thus implying that permanent employees have a higher likelihood of having more children. Being a permanent employee showed a weak negative correlation with work-family organizational support (*r* = -.13, *p* < .05). Control variables did not show any other correlation with study variables, thus we did not consider them in further analysis.

After controlling for parcels’ normality, checking whether Kurtosis and Skewness values were lower than ± 1.96 (Schaufeli, Bakker, & Salanova, 2006), we used Confirmatory Factor Analysis (CFA) to test the construct validity of all measures. We compared different nested models, where the first one was the one-factor model and the final one was the model with one factor for each study measure (in our case six factors). Chi square/degrees of freedom values and other goodness of fit indices were used to compare the different solutions.

The three measurement models tested were: a one-factor model (M1), a three-factor model (M2: work-family organizational support, the three mediators as a whole factor, the two outcomes as a whole factor), and a six-factor model (M3: encompassing the six study variables as separate factors). Table 2 shows fit indexes of each model.

**Table 2:** Competing Measurement Models

Model	χ^2^(*df*)	CFI	GFI	SRMR	RMSEA
M1	3538.9 (135)	.46	.43	.17	.25
M2	2390.25 (135)	.64	.58	.28	.21
M3	364.96 (120)	.96	.90	.04	.07

A remarkable improvement of all goodness of fit indexes can be observed between M1 and M2, and between M2 and M3. In particular, M3 showed satisfactory goodness of fit indexes (χ^2^ = 364.96, *df* = 120, CFI = .96, GFI = .90, SRMR = .04, RMSEA = .07) providing support for construct validity of all six study variables.

**Figure 2:**
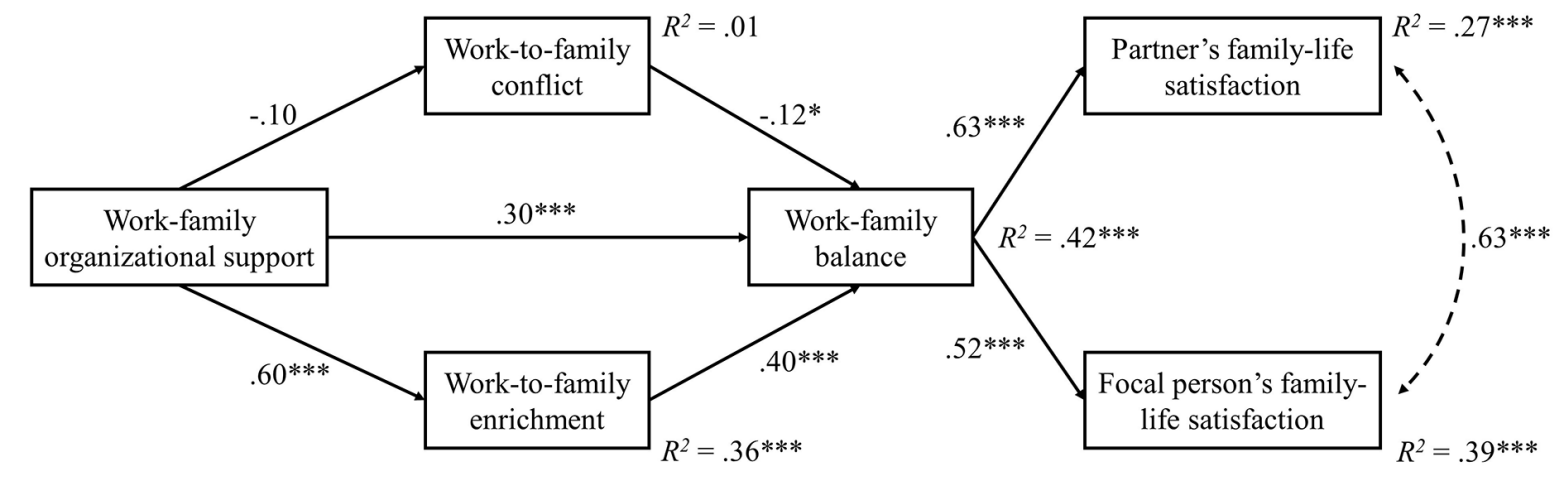
Structural model (standardized coefficients). *p < .05. ***p < .001.

Then we tested the hypothesized relationships through a structural model: two direct links from work-family organizational support to work-to-family enrichment and work-to-family conflict, two links from these two latter variables on one side, and work-family balance on the other, one link from work-family organizational support to work-family balance, and finally two links from work-family balance to family-life satisfaction and to partners’ family-life satisfaction as outcome variables. This model, depicted in Figure 2, showed adequate goodness of fit indexes: χ^2^ = 383.63, *df* = 127, CFI = .96, GFI = .90, SRMR = .05, RMSEA = .07.

Work-family organizational support positively predicted work-to-family enrichment (β = .60, *p* < .001) and work-family balance (β = .30, *p* < .001). Its negative relationship with work-to-family conflict was not significant (β = -.10). Work-family balance was negatively predicted by work-to-family conflict (β = -.12, *p* < .05) and positively by work-to-family enrichment (β = .40, *p* < .001). A significant indirect effect of work-family organizational support (β = .25, *p* < .001) towards work-family balance through work-to-family enrichment was present. Moreover, work-family balance positively predicted both focal person’s (β = .63, *p* < .001) and partner’s family-life satisfaction (β = .52, *p* < .001). Again, significant indirect effects of work-family organizational support towards focal person’s (β = .34, *p* < .001) and partner’s family-life satisfaction (β = .28, *p* < .001) through work-family balance were present. A significant positive correlation (*r* = .63, *p* < .001) between the two outcomes emerged. Finally, while the indirect effects from work-to-family conflict to the outcomes through work-family balance were not significant, significant indirect effects from work-to-family enrichment to both focal person’s (β = .25, *p* < .001) and partner’s family-life satisfaction (β = .21, *p* < .01) were present.

Explained variances for study variables were as follows: work-to-family conflict = 0.01, *ns*; work-to-family enrichment = 0.36, *p* < .001; work-family balance = 0.42, *p* < .001; focal person’s family-life satisfaction = 0.39, *p* < .001; partner’s family-life satisfaction = 0.27, *p* < .001.

## Discussion

The present paper, supported by spillover and crossover theories, investigated the intermediate role of work-family balance between WFOS, work-to-family enrichment and work-to-family conflict on the one side, and both partners’ family-life satisfaction, on the other. It contributed to the literature in different ways. In particular, it a) examined the role of WFOS, an organizational resource, in increasing work-family balance through the mediation of work-family enrichment and conflict; b) provided further evidence of the association between work-family enrichment, conflict and balance, investigating their differing effects on family-life satisfaction; c) analysed the relationship between work-family balance and the family-life satisfaction of both partners of dual-income families; d) tested the SCM, integrating the concept of work-family balance as an interpersonal exchange factor within the model.

Hypothesis 1 stated that a) work-to-family conflict would be negatively associated with work-family balance and b) work-to-family enrichment would be positively associated with work-family balance. Both hypotheses were confirmed, although, in accordance with the literature (Carlson et al., 2009), the association was stronger for work-to-family enrichment. Therefore, the study sustained evidence formerly presented in the literature (Carlson et al., 2009; Casper et al., 2018; Greenhaus & Allen, 2011) about the difference between work-family balance and the other two variables. It also highlighted the role of work-to-family enrichment and conflict as being determinants of work-family balance (Grawitch et al., 2013). Consistent with Carlson and colleagues (2009), work-family balance is achievable despite experiences of work-to-family conflict. Our results suggested that conflict between demands from the two roles, i.e. work and family, may decrease an individual’s ability to accomplish socially negotiated role-related responsibilities and expectations from the two sides. Yet this ability may be improved by the enrichment process, according to which positive experiences in one role, i.e., work, may increase the quality of life in the other, i.e., family. These findings highlighted how important promoting work-to-family enrichment is, as well as preventing and addressing work-to-family conflict, in supporting employees’ positive chances of meeting the requirements of both roles.

Hypothesis 2 introduced the organizational factor WFOS as a job resource, in order to investigate its association with both a) work-to-family conflict and b) work-to-family enrichment. Despite evidence suggesting a negative association between workplace social support and work-to-family conflict (e.g., Bellavia & Frone, 2005; Ghislieri, Gatti, Molino, & Cortese, 2017; Michel et al., 2011), the present study did not confirm this association; thus, Hypothesis 2a was rejected. Thus, WFOS, which represents the perception that managers and the organization are sensitive and supportive of employees’ need to balance work and family roles, does not seem effective enough to resolve the perception of incompatibility between family and work roles. It is necessary to investigate this relationship in order to understand whether more concrete work-family solutions are needed to decrease work-family conflict. In that sense, the role of colleagues should also be considered, since concerns about their potential resentment, the so called ‘work-family backlash’ (Ghislieri et al., 2017; Young, 1999), could prevent a person from fully benefitting from organizational and supervisory support. On the other hand, according to the SCM, Hypothesis 2b was confirmed, since a strong positive association between WFOS and work-to-family enrichment emerged. Support from the organization and supervisors can potentially improve the quality of work experience, in turn fostering work-to-family enrichment.

Hypothesis 3a suggested there would be a positive direct association between WFOS and work-family balance and the results supported this, confirming a positive relationship between social support and work-family balance (Ferguson et al., 2012). Moreover, a mediational role, suggested by the SCM, of both 3b) work-to-family conflict and 3c) work-to-family enrichment between WFOS (a job resource) and work-family balance (interpersonal exchange factor) was hypothesized. Consistent with indirect effects results and taking into consideration discussion of previous hypotheses, we can reject Hypothesis 3b and confirm 3c. In other words, WFOS has the potential to foster work-family balance directly as well as through work-to-family enrichment as a mediator. The positive association between work-family support received from the organization and the accomplishment of role-related responsibilities and expectations in the family and work domains can be considered an original contribution of this paper to work-family literature and, particularly, to the SCM. This contribution is worth addressing through interventions that will be discussed later.

Hypothesis 4a stated that work-family balance would be positively associated with the focal person’s family-life satisfaction and results confirmed that finding a balance between family and work roles may foster satisfaction with family-life. This supports previous studies that highlighted a similar pattern with different outcomes, such as conjugal happiness (Milkie & Peltola, 1999), marital satisfaction and time being invested in domestic chores and activities (Clarke et al., 2004). Hypothesis 4b, which investigated whether work-family balance could mediate the association between WFOS and family-life satisfaction, was also confirmed, supporting the spillover effect that was hypothesized in this study.

Finally, Hypothesis 5 considered the crossover effect, considering the partner’s family-life satisfaction as an outcome. Particularly, Hypothesis 5a suggested there would be a positive association between work-family balance and the partner’s family-life satisfaction and Hypothesis 5b introduced the intermediate role of work-family balance as a mediator between WFOS and the partner’s family-life satisfaction. The results confirmed both hypotheses supporting spillover-crossover effects that were assumed in this study. These findings provided evidence that the improvement of interpersonal exchange, as confirmed by the ability to accomplish role-related expectations by the focal-person, may crossover to the partner. This may result in greater satisfaction with his/her family-life, mediating the association of organizational support, as suggested by the SCM.

### Limitations and Future Studies

Among the limitations of this study, the first to be mentioned pertains to the cross-sectional design, which did not permit to infer cause-effect relationships between variables (Podsakoff, MacKenzie, & Podsakoff, 2012). Given the nature of the study’s hypotheses, future research should be carried out through longitudinal or diary approaches. Thus, relationships between considered constructs and influences over time could be further investigated, potentially providing stronger support for spillover and crossover effects. Regarding data, one strength of the study is that it involved both family partners; nevertheless, it used uniquely self-reporting data, raising the possibility that common method bias might have influenced results (Conway, 2002). Future studies might also include other-reported data, for instance supervisors or colleagues’ reports or cross-evaluations from partners. Moreover, objective data should also be considered, such as information about work-family policies and strategies adopted within organizations.

Another main limitation pertains to the convenience sampling procedure, since it precludes the generalizability of the results. However, it must be noted that no differences have been found comparing studies in the work-family field whose samples had been collected differently (Frone, 2003), and that convenience sampling is adequate when the aim is to obtain higher heterogeneity (Landers & Behrend, 2015). Moreover, the study involved only inter-gender couples, excluding same-gender ones. Research within specific organizational contexts or sectors would also be helpful to better understand dynamics related to work-family organizational support. The procedure itself might also present some limitations; despite the fact we provided instructions that requested each partner to fill out the questionnaire separately, we were unable to control or monitor if, or to what extent these instructions were adhered to. Additionally, although our sample may appear unbalanced with respect to the employment contract distribution given the higher percentage of permanent employees, it must be noted that it is consistent with the more general Italian situation (82.98%; Instituto Nazionale die Statistica [ISTAT], 2019).

Regarding study hypotheses, in future research, the crossover effect of family to the work domain (Lo Presti et al., 2016) could also be considered. Thus, it could be verified whether stressors experienced by one individual in the family domain may crossover to the work domain of his/her partner (Mauno & Kinnunen, 1999). Moreover, qualitative research in the field of crossover literature could support the understanding of the dynamics of transfer from one partner to the other.

### Conclusions and Practical Implications

Despite its limitations, this study contributed to work-family literature and may provide indications for organizational interventions. First, findings highlighted the role a supportive work-family culture plays in improving employees’ abilities to balance family and work domains and in enhancing the quality of their, and their partners’, family life. Informal and formal work-family balance strategies, interventions and policies should be developed within organizations, with constant monitoring of their impact, efficacy and effectiveness (Carlson et al., 2009). Among formal policies, organizations could consider flexible work designs (Hoeven & Zoonen, 2015) and welfare initiatives to facilitate daily administration.

Supervisors and colleagues may thrive through informal support. With this in mind, organizations could provide training opportunities to employees, particularly supervisors. These could improve knowledge of the positive implications of a family-supportive culture and highlight best practices to foster it. As the study showed, a good work-family balance may also positively relate to the family life satisfaction of both partners. As a result, managers and executives should create an environment that helps employees to achieve this balance, reducing conflict and enhancing enrichment. As for the promotion of work-family enrichment, it appears fundamental to encourage employees’ professional development through training, tutoring and mentorship in order to foster the development of resources and competencies that can be used in the family domain as well.

Finally, knowledge on crossover between partners may be used to support both employees and organizations (Bakker, Demerouti, & Schaufeli, 2005). In particular, employees could develop a better awareness about their own and their partners’ feelings, attitudes and perceptions regarding the intertwining of work and family lives. This knowledge could then be used to address the potential needs of the couples.
